# Single-molecule visualization of Pif1 helicase translocation on single-stranded DNA

**DOI:** 10.1016/j.jbc.2023.104817

**Published:** 2023-05-11

**Authors:** Mainak Mustafi, Youngho Kwon, Patrick Sung, Eric C. Greene

**Affiliations:** 1 Department of Biochemistry & Molecular Biophysics, Columbia University, New York, New York, USA; 2 Department of Biochemistry and Structural Biology, University of Texas Health Science Center at San Antonio, Texas, USA

**Keywords:** Pif1 helicase, SF1B helicase, single-molecule fluorescence, DNA curtains, ssDNA translocation, Pif1 processivity, ssDNA binding

## Abstract

Pif1 is a broadly conserved helicase that is essential for genome integrity and participates in numerous aspects of DNA metabolism, including telomere length regulation, Okazaki fragment maturation, replication fork progression through difficult-to-replicate sites, replication fork convergence, and break-induced replication. However, details of its translocation properties and the importance of amino acids residues implicated in DNA binding remain unclear. Here, we use total internal reflection fluorescence microscopy with single-molecule DNA curtain assays to directly observe the movement of fluorescently tagged *Saccharomyces cerevisiae* Pif1 on single-stranded DNA (ssDNA) substrates. We find that Pif1 binds tightly to ssDNA and translocates very rapidly (∼350 nucleotides per second) in the 5’→3′ direction over relatively long distances (∼29,500 nucleotides). Surprisingly, we show the ssDNA–binding protein replication protein A inhibits Pif1 activity in both bulk biochemical and single-molecule measurements. However, we demonstrate Pif1 can strip replication protein A from ssDNA, allowing subsequent molecules of Pif1 to translocate unimpeded. We also assess the functional attributes of several Pif1 mutations predicted to impair contact with the ssDNA substrate. Taken together, our findings highlight the functional importance of these amino acid residues in coordinating the movement of Pif1 along ssDNA.

Helicases are important enzymes that convert the chemical energy stored in ATP into mechanical work, allowing them to move along and manipulate nucleic acids. Nearly 100 helicases are encoded within the human genome, and these proteins participate in almost all aspects of nucleic acid metabolism (1, 2, 3, 4, 5, 6, 7, 8, 9, 10). Importantly, helicase mutations have been linked to numerous human diseases in which genomic instability, immunodeficiency, mental retardation, premature aging, and predisposition to cancer are common features (3, 11, 12, 13, 14, 15, 16, 17). The helicase superfamily 1 (Sf1) is one of the largest subgroups of helicases, and they are required for a range of cellular activities across all domains of life. Sf1 helicases can be subdivided into two classes called the Sf1a and Sf1b helicases, which move in opposite directions on nucleic acids (4, 7, 10, 18). Pif1 is an Sf1b helicase, and Pif1 homologs have been identified throughout biology ranging from bacteria to humans (19, 20, 21, 22).

*Saccharomyces cerevisiae* Pif1 was originally identified in a screen for mutations that changed the recombination frequency of tandemly arrayed repeats within mitochondrial DNA and was named after the resulting petite integration frequency phenotype (PIF1) (23). Pif1 was later identified in a screen for genes that affected telomere length, providing an indication that the helicase had a nuclear function in addition to its role in mitochondria (24). *S. cerevisiae* Pif1 has two start codons separated by 39 codons: translation from the first start codon generates a protein with a mitochondrial localization signal, while protein translated from the second start codon localizes to the nucleus (25). Additionally, while most organisms only encode one Pif1 family helicase, *S. cerevisiae* expresses two: Pif1 (97 kDa; 859 aa) and Rrm3 (81 kDa; 723 aa), which share 40% sequence identity within their helicase core domains (20). Pif1 also shares significant sequence homology to the bacterial protein RecD (26), including seven highly conserved helicase motifs and three additional motifs of unknown functions (termed named motifs A, B, and C) (26). Pif1 helicases also contain a Pif1 signature sequence (23 amino acid residues in length) which forms an α-helix and a turn that helps maintain a key phenylalanine residue (F71) in the appropriate position to assist with nucleic acid strand separation (27, 28, 29, 30, 31). Pif1 exhibits ATP-dependent helicase activity *in vitro* and can unwind double-stranded DNA (dsDNA) structures (32), G quadraplexes (33, 34, 35), and RNA–DNA hybrids (35, 36, 37) and translocates on nucleic acids in the 5’→3′ direction (38). Interestingly, Pif1 unwinds RNA-DNA hybrids better than duplex DNA, suggesting that it may participate in R-loop processing (37). Pif1 is known to participate in numerous aspects of genome maintenance, including telomere length regulation (24, 39); Okazaki fragment maturation (40, 41); assisting replication fork progression through difficult to replicate sites (33, 42, 43, 44); the maintenance of the replication fork barrier within ribosomal DNA (45); replication fork convergence during the completion of DNA synthesis (46); and DNA synthesis during break-induced replication (47, 48).

*S. cerevisiae* Pif1 has been widely studied by genetic, biochemical, and single-molecule techniques, which have all revealed important insights into Pif1 activities (reviewed in refs. (18, 25, 27)). To gain further insights into the mechanistic attributes of Pif1, single-stranded DNA (ssDNA) curtains together with total internal reflection fluorescence microscopy were used to visualize the helicase movement along different DNA substrates for hundreds of individual Pif1 molecules. Using this method, we could directly visualize GFP-tagged Pif1 as it moved along the single-strand DNA molecule bound by the ssDNA-binding protein replication protein A (RPA), allowing us to directly measure Pif1 velocity and processivity on RPA-ssDNA molecules. We show that Pif1 can rapidly displace RPA by pushing it along the ssDNA. A recent crystallography study was able to identify all the amino acid residues of Pif1 that are involved in binding to ssDNA (49). To functionally characterize these contacts, we introduced into Pif1 mutations predicted to disrupt each individual ssDNA contact. We demonstrate that mutations at 14 of 15 amino acid residues predicted to contact the ssDNA either partially or completely abrogate ATP hydrolysis and helicase activity in bulk assays, and that only four of the mutant proteins retain detectable translocation activity on RPA-ssDNA in our single-molecule analysis.

## Results

### Construction of fluorescently labeled Pif1

**Figure 1:**
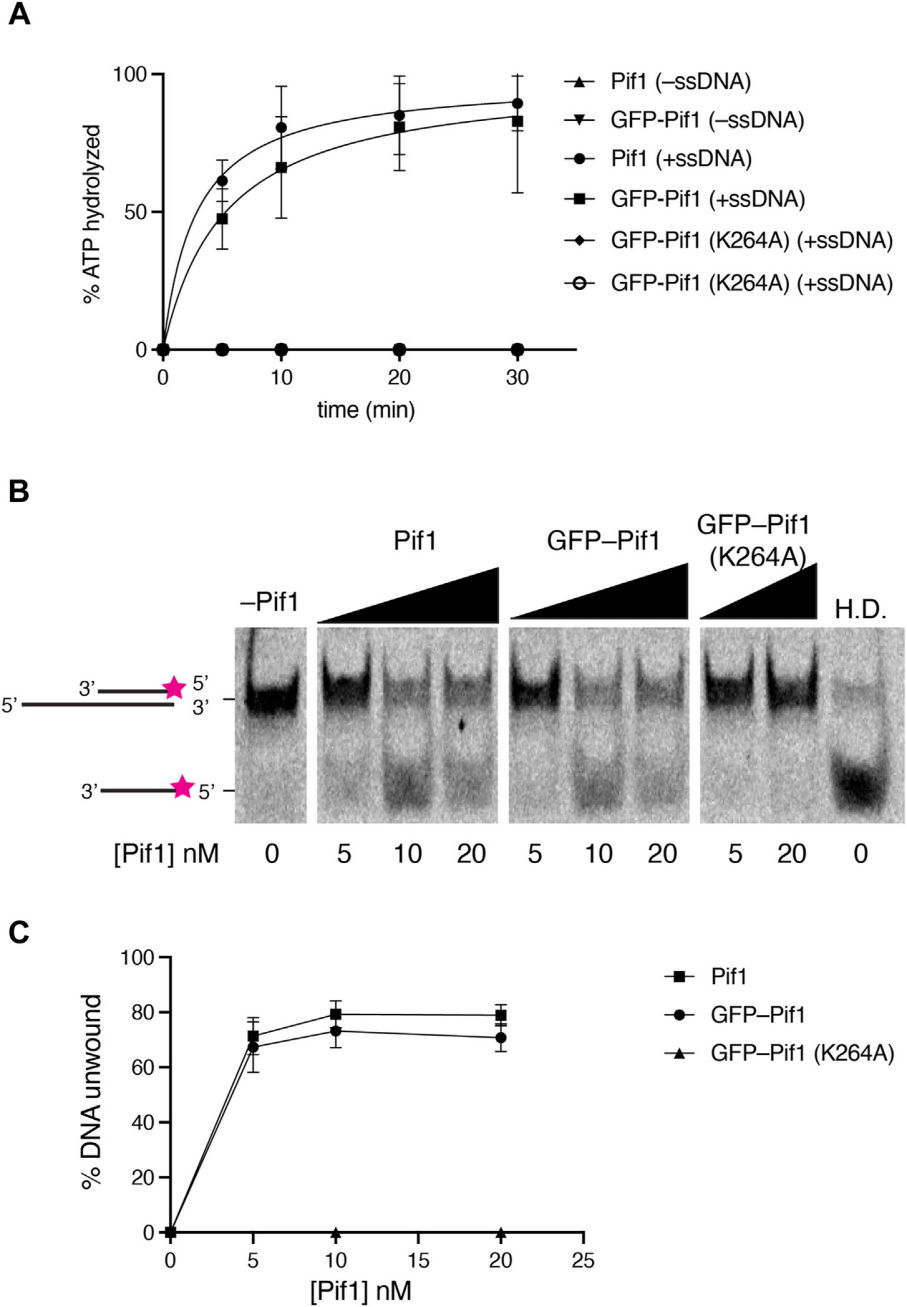
**Bulk characterization of GFP-tagged Pif1 and comparison to unlabeled Pif1**. *A*, fraction of ATP hydrolyzed over time for WT Pif1 (unlabeled) and GFP-tagged Pif1. All reactions were conducted in the presence of ssDNA, and the GFP-Pif1 K264A ATP hydrolysis–deficient mutant was included as a negative control. *B*, comparison of DNA unwinding activities for Pif1, GFP-Pif1, and GFP-Pif1 K264A in assays using a fluorescently labeled oligonucleotide-based substrate with a 40-nt 5′ overhang and a 40-bp segment of flanking dsDNA. The migration positions of the unreacted and unwound DNA fragments within the gel are *highlighted*. A heat denatured (H.D.) control is also shown. *C*, graph depicting the quantitation of the helicase activity (% DNA unwound) for Pif1, GFP-Pif1, and GFP-Pif1 K264A. Each experiment was conducted in triplicate, and error bars represent the standard error of the mean. Note that the panels in *B* were from the same gel but were re-ordered in the figure for clarity.

We sought to prepare a fluorescently labeled Pif1 for our total internal reflection fluorescence microscopy–based DNA curtain assays. For this, we generated an expression construct in which GFP was fused to the N terminus of *S. cerevisiae* Pif1 (see Experimental procedures). We examined GFP–Pif1 in bulk assays to compare its levels of ATPase and helicase activities to those of unlabeled Pif1. As negative control, we generated a version of GFP–Pif1 in which lysine residue 264 within the Walker A ATP-binding motif has been mutated to alanine (K264) (39). The GFP–tagged version of Pif1 retained wildtype levels of ATPase activity in the presence of M13 ssDNA (Fig. 1*A*) and helicase activity in assays using a 40–base pair (bp) dsDNA fragment with a 40–nucleotide (nt) 5′ ssDNA overhang (Fig. 1, *B* and *C*). As expected, we did not detect any ATPase or helicase activity for GFP–Pif1–K264A (Fig. 1, *A*–*C*). These results indicate that GFP–tagging of Pif1 has little or no impact on its enzymatic activities.

### DNA dependency of Pif1 ATPase activity

Before beginning the single-molecule experiments, we first sought to determine how the ATPase activity of Pif1 and GFP–Pif1 is affected by ssDNA as compared to dsDNA. These assays confirmed that Pif1 and GFP–Pif1 do not hydrolyze ATP in the absence of a DNA substrate (Fig. S1, *A* and *B*) and also showed a higher ATP hydrolysis rate with ssDNA compared to dsDNA; the ATP hydrolysis rates at 2.5 mM ATP with ssDNA was 5.2 ± 2.2 μM sec^–1^ for unlabeled Pif1 and 5.2 ± 3.6 μM sec^–1^ for GFP–Pif1 in reactions with ssDNA, whereas the rates for ATP hydrolysis was 1.7 ± 0.8 μM sec^–1^ for unlabeled Pif1 and 1.6 ± 1.0 μM sec^–1^ for GFP–Pif1 in reactions with dsDNA (Fig. S1).

### Pif1 ATP hydrolysis activity is inhibited by RPA

We consider the ATP hydrolysis assays to be a proxy for Pif1 translocation on DNA, thus our data suggest that Pif1 may exert more robust translocation on ssDNA compared to dsDNA. However, naked ssDNA does not exist more than transiently under physiological conditions but instead becomes bound by the highly abundant RPA, an essential ssDNA-binding protein that binds ssDNA with affinities on the order of ∼10^–9^ to 10^–10^ M and fulfils an essential role in all nucleic acid processing reactions entailing a ssDNA intermediate (50, 51). In addition, from a technical perspective, our ssDNA curtain measurements rely upon RPA (or a GFP– or mCherry–tagged version of RPA) to eliminate secondary structure in the DNA and to prevent entropic collapse of the ssDNA, thus allowing ssDNA to be extended by hydrodynamic force and observed by total internal reflection microscopy (52). Next, we asked whether and how Pif1 may act upon ssDNA in the presence of RPA.

**Figure 2:**
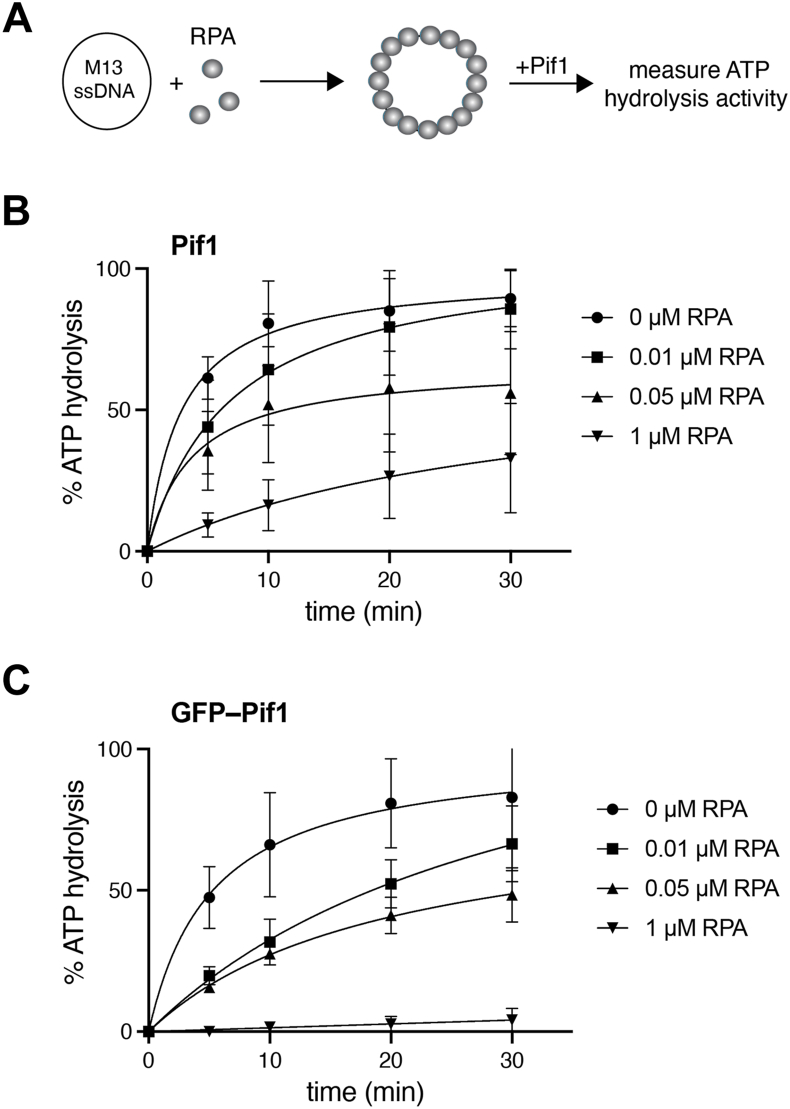
**ATP hydrolysis activity of unlabeled Pif1 and GFP–Pif1 in the presence of RPA-ssDNA**. *A*, schematic illustrating the assay to assess the effect of RPA on GFP-Pif1 ATP hydrolysis activity. *B*, fraction of ATP hydrolyzed over time by unlabeled Pif1 presented at varying concentrations of RPA. *C*, fraction of ATP hydrolyzed over time by GFP-Pif1 presented at varying concentrations of RPA. Each experiment was conducted in triplicate, and error bars represent the standard error of the mean. RPA, replication protein A; ssDNA, single-stranded DNA.

*S. cerevisiae* RPA binds to ssDNA in multiple modes, yielding occluded site sizes of 18 to 20 nts at low salt and 26 to 28 nts at high salt (53). Under the buffer conditions used for our ATPase assays with 60 mM KCl, we presume that RPA predominantly operates in the 18 to 20 nucleotide-binding mode. These assays used phage M13 (7249 nts) as the ssDNA substrate at a final concentration of 1.5 μM nts, corresponding to ∼360 RPA binding sites of 20 nts in length (or ∼75 nM binding sites). The assays contained either 0.01, 0.05, or 1.0 μM RPA, as indicated (Fig. 2). These experiments revealed that GFP-Pif1 ATPase activity decreases with increasing concentrations of RPA, yielding a 67% (*k*_*obs*_ = 1.73 ± 0.5 μM/sec) and 64% (*k*_*obs*_ = 1.88 ± 0.65 μM/sec) reduction in the observed rate of ATP hydrolysis (*k*_*obs*_) for reactions with 0.01 and 0.05 μM RPA, respectively, compared to reactions with no RPA (*k*_*obs*_ = 5.2 ± 3.6 μM/sec; Fig. 2). Notably, ATP hydrolysis by GFP-Pif1 was greatly affected by the supersaturating concentration of RPA (1.0 μM), yielding a 99% reduction (*k*_*obs*_ = 0.06 ± 0.1 μM/sec; Fig. 2).

### Translocation of Pif1 on ssDNA and RPA removal

**Figure 3:**
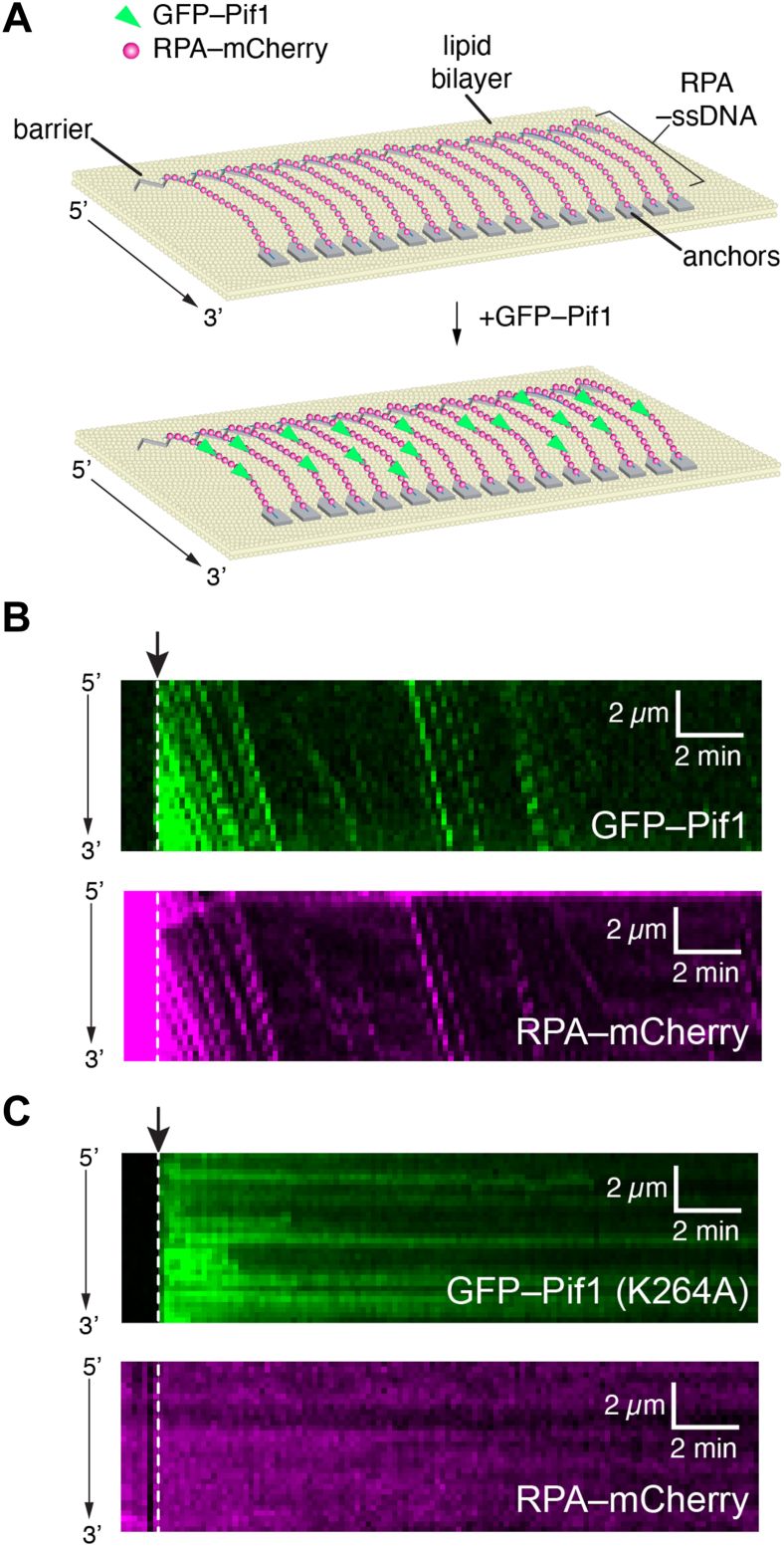
**Single-molecule ssDNA curtain assays for visualizing GFP-Pif1 translocation on RPA-ssDNA**. *A*, schematic illustration of an ssDNA curtain anchored through a 5′ biotin-streptavidin linkage to a supported lipid bilayer on the surface of a microfluidic sample chamber and aligned at chromium (Cr) barriers to lipid diffusion where the downstream sections of the ssDNA are anchored to exposed Cr anchor points. The ssDNA is coated with RPA-mCherry (*magenta*), and the GFP-Pif1 is shown in *green*. *B*, kymographs showing the mCherry and GFP channels for a single ssDNA molecule. At the beginning of the kymograph, the ssDNA is coated with RPA-mCherry (*magenta*), and GFP-Pif1 (*green*) was injected into the sample chamber; the injection time point is indicated with an *arrowhead*. *C*, kymographs showing the mCherry and GFP channels for a single ssDNA molecule. At the beginning of the kymograph, the ssDNA is coated with RPA-mCherry (*magenta*), and the ATP hydrolysis–deficient mutant GFP-Pif1 K264A (*green*) was injected into the sample chamber; the injection time point is indicated with an *arrowhead*. RPA, replication protein A; ssDNA, single-stranded DNA.

We next sought to determine whether we could visualize GFP–Pif1 translocation on double–tethered ssDNA curtains (Fig. 3*A*) (54, 55). Herein, the ssDNA was anchored to a supported lipid bilayer on a flow cell surface through a biotin–streptavidin linkage and then pushed into chromium barriers to lipid diffusion that were deposited onto the flow cell surface by electron beam lithography. RPA–mCherry (0.1 nM) was then injected into the sample chamber to unravel the ssDNA to allow the downstream end of the ssDNA to be anchored to exposed chromium anchors *via* nonspecific surface absorption (Fig. 3*A*). A 50 μl aliquot of GFP–Pif1 (0.5 nM final concentration) was then injected into the flow cell at a constant rate of 0.2 ml/min in buffer that lacked any additional RPA–mCherry, and imaging was conducted at one frame per 10 s for a total of 30 min (Fig. 3*B*). The analysis revealed that GFP–Pif1 quickly associates with RPA–ssDNA molecules and begins translocating in the 5’→3′ direction (Fig. 3*B*), which is consistent with expectations for a helicase of the Sf1b family and for Pif1 specifically (38). Experiments testing the ATPase defective GFP–Pif1–K264A revealed its extensive association with the RPA–mCherry–bound ssDNA but yielded no evidence for 5’→3′ translocation (Fig. 3*C*). Thus, the DNA curtain methodology is well suited to interrogating the ATP–dependent 5’→3′ translocation of GFP–Pif1 on ssDNA molecules.

Interestingly, GFP–Pif1 binding and movement coincided with a rapid loss of bound RPA–mCherry signal from the ssDNA, suggesting that Pif1 was stripping RPA–mCherry from the ssDNA (Fig. 3*B*). Moreover, inspection of the resulting kymographs revealed that at later reaction time points, when the number of RPA–mCherry molecules bound to the ssDNA was very low, clear evidence could be observed of GFP-Pif1 rapidly pushing isolated RPA–mCherry molecules for long distances (tens of kilonucleotides) along the ssDNA substrate (Fig. 3*B*). The finding that Pif1 could push RPA along ssDNA is consistent with prior single-molecule Fluorescence Resonance Energy Transfer or smFRET studies of Pif1 activities (56, 57). The apparent difference between the bulk experiments, where RPA inhibits Pif1 ATP hydrolysis activity, and the single-molecule experiments, where Pif1 can strip RPA from the ssDNA, is likely due to the fact that free RPA is always present in solution in the bulk experiments and thus capable of re-binding the ssDNA after displacement, whereas free RPA is not present in the single-molecule experiments and can be flushed from the sample chamber after removal from the ssDNA.

**Figure 4:**
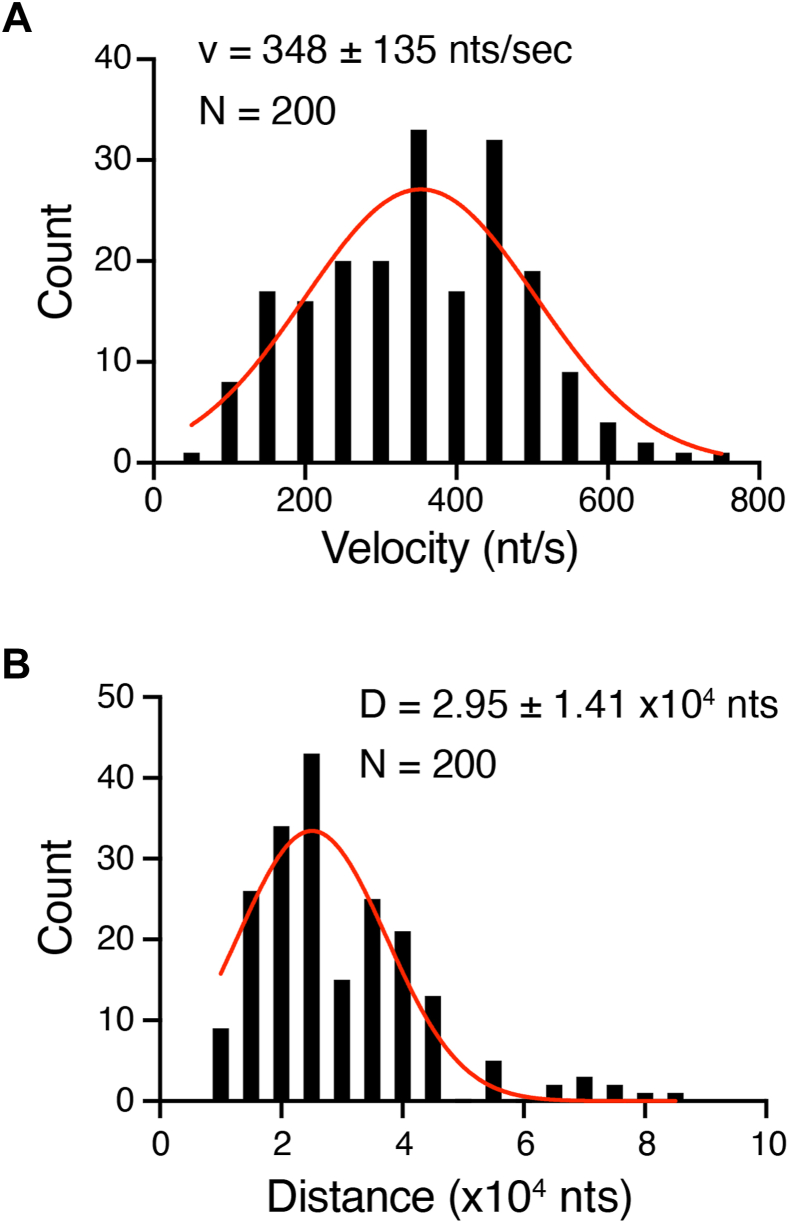
**Characteristics of GFP-Pif1 translocation on RPA–ssDNA**. *A*, graph showing the distribution of velocities observed for GFP-Pif1 translocation on single molecules of RPA-ssDNA. A Gaussian fit to the data (*red curve*) yields a mean velocity of 348 ± 135 nt/sec (N = 200). *B*, graph showing the distribution of distances traveled observed for GFP-Pif1 translocation on single molecules of RPA-ssDNA. A Gaussian fit to the data (*red curve*) yields a mean distance of 2.95 ± 1.41 × 10^4^ nt (N = 200). RPA, replication protein A; ssDNA, single-stranded DNA.

Analysis of kymographs for GFP–Pif1 translocation activity on the RPA–bound ssDNA substrates revealed a mean translocation velocity of 348 ± 135 nts per second (N = 200) and mean observed translocation distance of 29,500 nts (N = 200) (Fig. 4, *A* and *B*). Note that our reported value of 29,500 nts for the translocation distance reflects only the distance that we observe the molecules travel rather than an absolute value for Pif1 processivity. Two caveats must be considered when interpreting this value, the first being that it is formally possible that we are missing short distance transient events, which could contribute to an overestimate for the processivity, and the second being that the reported observed distance is comparable to the length of the ssDNA substrate tethered between the barriers and pedestals on the flow cell surface, which could lead to an underestimate for the processivity. Regardless, we conclude that Pif1 exhibits robust ATP hydrolysis–dependent ssDNA translocation activity and can translocate for very long distances in our assays.

### Mutation of Pif1 ssDNA–binding residues

**Figure 5:**
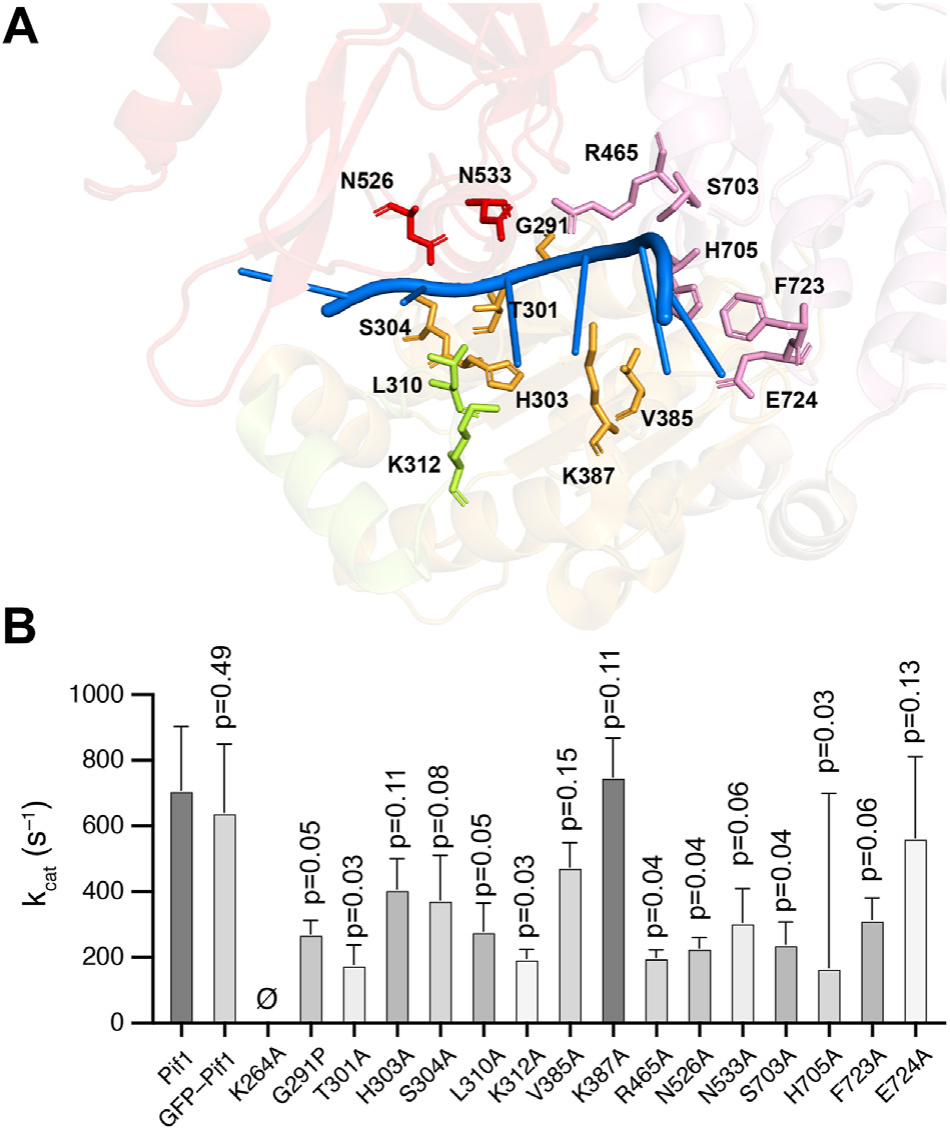
**Design of Pif1 mutants and their ATP hydrolysis activity**. *A*, image showing the ssDNA-binding cleft of *S. cerevisiae Pif1* (PDB ID: 5O6B (49)). Amino acid residues that make contact with the ssDNA are highlighted. *B*, graph displaying relative ATP hydrolysis activity (*k*_cat_) for WT Pif1, GFP-Pif1, GFP-Pif1 K264A, and all of the indicated mutant proteins. All *p* values correspond to statistical comparison with the WT dataset. Also refer to Table S1. ssDNA, single-stranded DNA.

The structure of *S. cerevisiae* Pif1 bound to ssDNA has been solved by X-ray crystallography (49), which reveals amino acid residues that directly contact ssDNA (Fig. 5*A*). These amino acid residues span domains 1A, 1B, 2A, and 2B of Pif1 and likely play important roles in mediating the transduction of chemical energy derived from ATP hydrolysis to mechanical energy allowing the protein to translocate on ssDNA. To further understand the structural basis for Pif1 ssDNA translocation activity, we constructed a total of 15 single mutations in these ssDNA–binding amino acid residues, namely, G291P, T301A, H303A, S304A, L310A, K312A, V385A, K387A, R465A, N526A, N533A, S703A, H705A, F723A, and E724A (Fig. 5*A*). In most instances, the amino acid residues of interest were changed to alanine, except for glycine 291 which was instead mutated to proline. These Pif1 mutants were expressed as GFP-fusion proteins and purified from *Escherichia coli* and then subjected to bulk biochemical and single-molecule analyses, as described below.

### ATPase activity of Pif1 mutant proteins

As an initial assessment, each of the mutant proteins was tested for ATPase activity. ATP hydrolysis assay was performed by incubating 10 nM Pif1 with M13 ssDNA (7249 nts in length, 1.5 μM nts final concentration) and [γ-^32^P]-ATP. Reactions were incubated at 30 °C, and aliquots removed at 5-, 10-, 20-, and 30-min time points and terminated by addition of an equal volume of 50 mM EDTA. The quenched reactions were then resolved by thin layer chromatography (TLC), and ATP hydrolysis was quantified by phosphorimaging of dried TLC plates. Values for *V*_max_, K_m_, and *k*_cat_ were determined and compared to WT Pif1 and the Walker A Pif1 mutant K264A (Fig. 5*B* and Table S1). Analysis of WT Pif1 yielded a *k*_cat_ value of 646.1 ± 125.7 s^–1^, whereas no ATP hydrolysis was detected for Pif1 K264A, as anticipated (Fig. 5*B* and Table S1). Most of the mutant proteins showed reduced ATPase activity compared to WT Pif1 (Fig. 5*B* and Table S1). Notably, the least affected mutant was Pif1 V385A, which yielded a 26% decrease in *k*_cat_ (475.2 ± 74.4 s^–1^) which was statistically indistinguishable from WT Pif1 activity (*p* = 0.15; Table S1), whereas the most greatly affected mutant Pif1 H705A yielded a 74% decrease in *k*_cat_ (167.6 ± 532.3 s^–1^; *p* = 0.03; Fig. 5*B* and Table S1). The remaining mutants fell between these two values with the exception of Pif1 K387A which instead displayed a 35% increase in *k*_cat_ (874.7 ± 115.2 s^–1^) compared to WT Pif1 (Fig. 5*B* and Table S1), although it should be noted that this difference was not statistically significant (*p* = 0.11; Table S1).

### Bulk biochemical ssDNA-binding activity of Pif1 mutants

**Figure 6:**
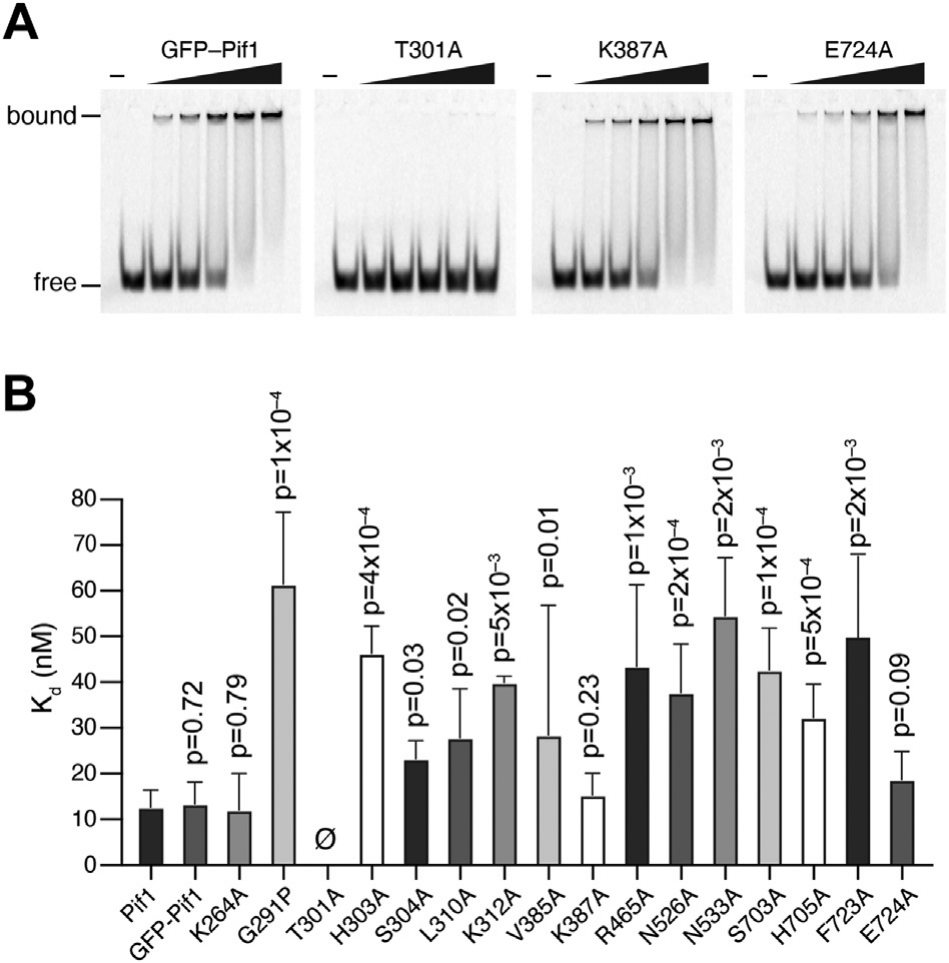
**ssDNA-binding activity of Pif1 mutant proteins.***A*, examples of electrophoretic mobility shift assays (EMSA) GFP-Pif1 and the GFP-tagged Pif1 mutants T301A, K387A, and E724A in reactions with a 40-nt ssDNA substrate. See Fig. S2 for EMSAs with all additional mutant proteins and Table S1 for K_d_ values. *B*, quantitation of the binding data for all Pif1 proteins and mutants. Each experiment was conducted in triplicate and error bars represent the standard deviation. All *p* values correspond to statistical comparison with the WT dataset. ssDNA, single-stranded DNA.

We used the electrophoretic mobility shift assay for probing the ssDNA-binding properties of the Pif1 mutants. For this, Pif1 (3.125, 6.25, 12.5, 25, and 50 nM) was incubated with a 40-nt ssDNA (5 nM) that was labeled at the 5′ end with the fluorophore in buffer containing 35 mM Tris–HCl [pH 7.5], 1 mM DTT, 5 mM MgCl_2_, 60 mM KCl, and 0.1 mg/ml BSA but no ATP. Following a 30-min incubation at 30 °C, the free ssDNA and nucleoprotein complexes were resolved in 8% polyacrylamide gels (Figs. 6*A* and S2), and the Kd values obtained from the binding data. These experiments revealed a binding affinity of 12.62 nM and 12.06 nM for WT Pif1 and Pif1 K264A, respectively (*p* = 0.92; Table S1). Interestingly, we could detect no ssDNA-binding activity for the mutant protein Pif1 T301A (Fig. 6, *A* and *B*), even though this mutant exhibited weak but detectable ATP hydrolysis activity (Fig. 5*B* and Table S1). Given that ATP hydrolysis by WT Pif1 is DNA dependent, it is possible that for this particular mutant, the residual ATP hydrolysis activity is somehow decoupled from ssDNA binding. Alternatively, and perhaps more likely, it is possible that the reduced ATP hydrolysis activity observed for Pif1 T301A stems from a much weakened affinity for ssDNA binding that could not be detected by electrophoretic mobility shift assay. Apart from Pif1 T301A, all remaining mutants exhibited ssDNA-binding activity (Fig. 6*B* and Table S1). The weakest binding mutants were Pif1 G291A and Pif1 F723A, which yielded Kd values of 61.41 ± 15.80 nM (*p* = 0.04) and 55.58 ± 18.54 (*p* = 0.06), respectively (Fig. 6*B* and Table S1). Note that even through these mutant proteins bind ssDNA more weakly than WT Pif1, we still consider Kd values on the order of 40 to 60 nM to reflect relatively high ssDNA-binding affinity.

### Bulk biochemical helicase activity of Pif1 mutants

**Figure 7:**
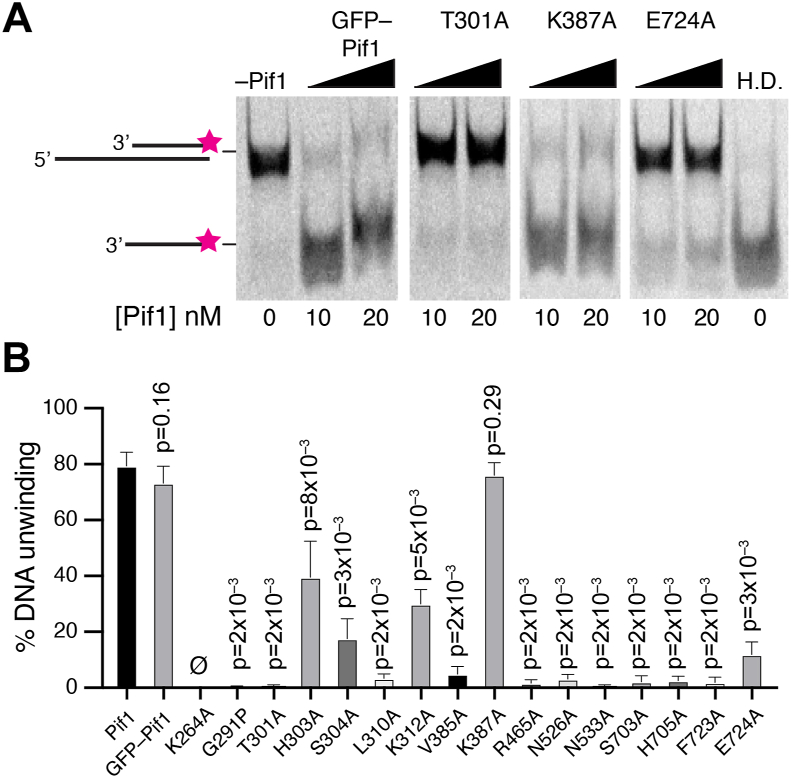
**Helicase activity of Pif1 mutant proteins.***A*, examples of gels showing the DNA unwinding activity for GFP-Pif1, and the GFP-tagged Pif1 mutants T301A, K387A, and E724A. A heat denatured (H.D.) control is also shown. See Fig. S3 for examples of helicase assays with all additional Pif1 mutants. *B*, quantitation showing the fraction of ssDNA unwound (% unwound) in 3-min reactions with each of the indicated Pif1 proteins and mutants. See Table S1 for data values for the quantitation. Each experiment was conducted in triplicate, and error bars represent the standard error of the mean. All *p* values correspond to statistical comparison with the WT dataset.

We used biochemical helicase assays to assess the ability of the Pif1 mutant proteins to unwind duplex DNA. For this assay, we annealed an 80-nt ssDNA to a complementary 40-nt ssDNA labeled at its 5′ terminus with an Alexa 647 dye to generate a substrate with a 40-base pair region of duplex DNA flanked by a 40-nt 5′ overhang. Pif1 was then tested with 10 nM of DNA substrate in buffer containing 2 mM ATP plus 40 nM RPA, and reactions were incubated at 30 °C for 3 min, deproteinized, and resolved in an 8% polyacrylamide gel and imaged (Figs. 7*A* and S3). The results revealed that many of the mutants proteins are greatly impaired for DNA unwinding as compared to WT Pif1. This was true even for the mutants that retain ATPase and ssDNA-binding activities (Figs. 7, *A* and *B* and S3). For WT Pif1, approximately 79% of the input dsDNA substrate was unwound during the 3-min reaction, whereas the most strongly impacted mutants showed ≥94% reduction in DNA unwinding activity, and these include the G291P, T301A, L310A, V385A, R465A, N526A, N533A, S703A, H705A, F723A, and E724A mutants (Fig. 7*B* and Table S1). For example, the most strongly affected mutant, Pif1 G291P, exhibited a 99.6% reduction in DNA unwinding activity, which is in concordance with its reduced ATPase (*k*_cat_ 271 ± 41.6 s^–1^) and ssDNA-binding activities (K_d_ = 61.41 ± 15.8 nM; Table S1). Similarly, Pif1 T301A exhibited a 99.2% reduction in DNA unwinding activity, also consistent with its reduced ATPase (*k*_cat_ 178 ± 60.5 s^–1^) and ssDNA-binding activities (undetected ssDNA binding; Table S1). In contrast, Pif1 K387A was statistically indistinguishable from WT Pif1 for dsDNA unwinding, and its ATP hydrolysis and ssDNA-binding properties were similarly unaffected (Fig. 7*B* and Table S1). Mutants with more intermediate effects included Pif1 H303A, S304A, and K312A, which yielded a 50%, 78%, and 62% reduction in dsDNA unwinding activity, respectively (Fig. S3 and Table S1). We note that even though these latter mutants are less impaired for ATP hydrolysis or ssDNA binding compared to other mutants that exhibit a more severe DNA unwinding deficiency (Table S1), they are nonetheless still significantly impaired for helicase activity.

### Single-molecule ssDNA translocation characteristics

**Figure 8:**
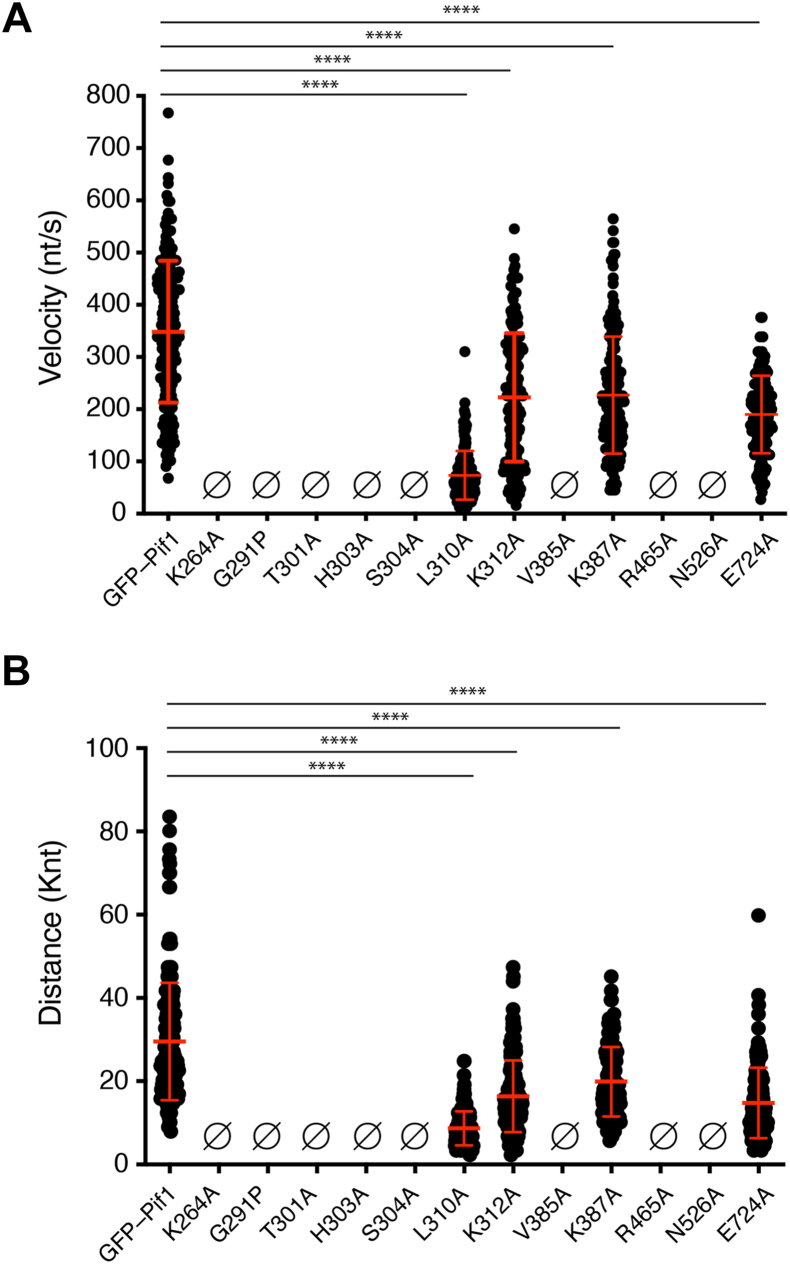
**Single-molecule translocation characteristics of Pif1 mutants on RPA-ssDNA**. *A*, graph showing the distribution of velocities observed for GFP-Pif1 proteins and mutants translocation on single molecules of RPA-ssDNA. *Red bars* highlight the mean and standard deviation of each dataset. *B*, graph showing the distribution of distances traveled for GFP-Pif1 proteins and mutants translocating on single molecules of RPA-ssDNA. *Red bars* highlight the mean and standard deviation of each dataset. All *p* values correspond to statistical comparison with the GFP-Pif1 dataset, and the “∗∗∗∗” is used to indicate the *p* values are <1 × 10^–5^. Note that the datasets for GFP-Pif1 are reproduced from here from Fig. S4 for comparison. See Table S2 for additional information. RPA, replication protein A; ssDNA, single-stranded DNA.

Next, we tested each of the mutant in the DNA curtain assay to assess their ability to translocate on RPA-coated ssDNA. Interestingly, for 11 of the 15 mutant proteins, we were unable to detect any evidence for their translocation on RPA-ssDNA, and they instead behaved most similarly to the ATPase defective mutant Pif1 K264A (Fig. 8 and Table S2). This class of nontranslocating Pif1 mutants included G291P, T301A, H303A, S304A, V385A, R465A, N526A, N533A, S703A, H705A, and F723A. This result highlights the functional significance of the amino acid residues in question with respect to Pif1 ssDNA translocation characteristics. Several of these Pif1 mutants that lack translocation activity on RPA-ssDNA also exhibited significant defects for ATP hydrolysis, ssDNA binding, and dsDNA unwinding activities, and these included G291P, T301A, R465A, N526A, N533A, S703A, H705A, and F723A (Table S1). Interestingly, even though Pif1 H303A, S304A, and V385A all retain a significant level of ATPase activity and exhibit more moderate defects in ssDNA binding and dsDNA unwinding, all three of these mutants are defective for translocation on RPA-ssDNA (Fig. 8 and Table S1).

Only four of the Pif1 mutants retained detectable, albeit compromised, translocation on RPA-ssDNA in DNA curtain analysis (Fig. 8 and Table S2). These included Pif1 L310A, K312A, K387A, and E724A. As indicated above, GFP-Pif1 translocated along the RPA-ssDNA at a rate of 348 ± 135 nts/sec for an average distance of 29.5 ± 14.1 knts (Figs. 4, 8 and Table S2). The most affected mutant protein was Pif1 L310A, which exhibited a 79% reduction in translocation velocity and a 71% reduction in translocation distance compared to WT Pif1, yielding values of just 73 ± 47 nts/sec and 8.7 ± 4.1 knts for velocity and distance, respectively (Fig. 8 and Table S2). In agreement with this finding, Pif1 L310A also exhibited defects in ATP hydrolysis (57% reduction compared to WT), ssDNA binding (Kd = 27.8 nM), and DNA unwinding (96% reduction compared to WT; Table S1). The remaining three mutant proteins, Pif1 K312A, K387A, and E724A, exhibited more moderate reductions in translocation velocity compared to Pif1 L310A, yielding values of 223 ± 123 nts/sec (36% reduction), 219 ± 112 nts/sec (37% reduction), and 190 ± 74 nts/sec (45% reduction), respectively (Fig. 8*A* and Table S2). Similarly, these three mutants exhibited more moderate reductions in translocation distance, yielding values of 16.4 ± 8.6 knts (44% reduction), 19.9 ± 8.3 knts (33% reduction), and 14.8 ± 8.4 knts (50% reduction) for Pif1 K312A, K387A, and E724A, respectively (Fig. 8*B* and Table S2). These three mutant proteins also exhibited a range of behaviors in the bulk biochemical assays, but none was as defective in these assays as the class of Pif1 mutants that showed no translocation activity in the single-molecule assays. For example, the K312 mutant exhibited a 70% reduction in ATPase activity, an ssDNA binding affinity of 40 nM, and a 62% reduction in helicase activity (Table S1). In contrast, the K387A mutant exhibited robust ATP hydrolysis activity, an ssDNA-binding affinity of 29 nM, and helicase activity that was comparable to WT Pif1 (Table S1). Finally, the E724A mutant exhibited robust ATP hydrolysis activity and an ssDNA-binding affinity of 19 nM, but the helicase activity was 85% lower than WT Pif1 (Table S1).

## Discussion

Pif1 is a conserved helicase that is essential for the maintenance of genome integrity and participates in numerous DNA transactions. Our findings reveal that *S. cerevisiae* Pif1 is a robust ATP-dependent ssDNA translocase with a 5′ to 3′ directionality and also exhibits a translocation processivity higher than previously realized. Our findings also reveal that Pif1 can efficiently strip RPA from ssDNA and push isolated RPA molecules over long distances. Our mutational analysis of key amino acid residues within the ssDNA-binding cleft provides insights into their functional importance.

### Mutational analysis of the Pif1 ssDNA-binding cleft

We have analyzed the impact of Pif1 mutations in amino acid residues shown to contact ssDNA within the Pif1–ssDNA crystal structures (49) by bulk biochemical and single-molecule assays. The crystal structure of *S. cerevisiae* Pif1 reveals a 6-nt binding cleft, and there are three available structures with three different oligonucleotide sequences bound with within this cleft, including 5′-TTTTTT-3′, 5′-GGGTTT-3′, and 5′-TTTGGG-3’ (49). The binding cleft itself is comprised of approximately 15 amino acid residues that contact the ssDNA, although the exact number of contacts varies for the different ssDNA sequences (49). It is notable that mutations at 14 of 15 amino acid residues predicted to contact the ssDNA either partially or completely abrogate helicase activity in bulk assays and also abrogate the translocation activity on RPA-ssDNA for all but four of the mutants.

Eight of the mutants that were tested did not exhibit any ssDNA translocation activity in the DNA curtain assay and were also significantly compromised for biochemical activities in bulk assays. These mutants spanned domains 1A, 2A, and 2B and included G291P, T301A, R465A, N526A, N533A, S703A, H705A, and F723A, all of which exhibited significant defects in ATPase, ssDNA-binding, and helicase activities. Notably, each of these amino acid residues is expected to contact the phosphate backbone of the bound ssDNA, and these contacts are present within all three *S. cerevisiae* Pif1–ssDNA crystal structures highlighting the general nonspecific nature of these phosphate backbone interactions (49). The T301A mutation appears to uniquely affect Pif1 ssDNA-binding activity, and this was the only mutant for which we were unable to detect ssDNA binding.

All four of the mutants that exhibited detectable translocation activity (L310A, K312A, K387A, and E724A) contact the nucleobases rather than the phosphate backbone, and three of these amino acid side chains only make contacts with ssDNA in one of the three available DNA-bound crystal structures (K312, K387, and E724): K312 contacts the third guanosine, K387 contacts the second guanosine, and E724 also contacts the second guanosine only in the cocrystal structures with the ssDNA sequence 5′-GGGTTT-3′ within the Pif1-binding cleft (49). These contacts were not observed in the crystal structures with the ssDNA sequences 5′-TTTTTT-3′ or 5′-TTTGGG-‘3 within the Pif1-binding cleft (49). Of these amino acid residues, only the L310 interaction is observed in all three crystal structures, and it interacts with two adjacent bases, either T-T or G-G at the fourth and fifth positions (numbered from the 5’ end) within the aforementioned sequences (49). Interestingly, mutation of L310 to alanine (L310A) causes a reduction in translocation velocity to just 73 nt/sec, whereas the K312A, K387A, and E724A mutants exhibit more modest effect with observed translocation velocities of 223, 219, and 190 nt/sec. These findings suggest that mutations of amino acid residues that make nucleobase contacts are generally more well-tolerated compared to amino acid residues that make contacts with the phosphate backbone (with one exception, see below). Moreover, mutation of L310, which makes nucleobase contacts in all three cocrystal structures, is more deleterious than mutation of either K312, K387, or E724 all of which appear to contact DNA nucleobases only in specific G-rich sequence contexts (49).

Interestingly, three of the mutants (H303A, S304A, and V385A) showed relatively robust levels of ATP hydrolysis with *k*_cat_ values ranging from 376 to 475 s^–1^, which are statistically distinguishable from that of WT Pif1. However, these three mutants are compromised for ssDNA-binding and helicase activity ranging from 50% to 94% the WT level, and none of the three exhibited translocation activity on RPA-ssDNA in single-molecule DNA curtain analysis. These three amino acid residues reside with domain 1A and contact the ssDNA in all three Pif1-ssDNA crystal structures: H303 interacts with a deoxyribose ring at the fourth position (numbered from the 5′ end) and is thought to play a role in helping to discriminate between ssDNA and ssRNA; S304 contacts the phosphate backbone at the 3′ most edge of the binding cleft; and V385 makes contacts with two adjacent nucleobases, either T-T or G-G, in the reported structures (49). Given that the H303A, S304A, and V385A mutants are all competent for ATP hydrolysis while severely compromised for helicase and translocation activities, we speculate that these amino acid residues may contribute to a clutch-like mechanism, the disruption of which prevents Pif1 from efficiently moving along a ssDNA substrate, perhaps similar to what we have reported for W325 of AdnB within the AdnAB helicase complex (58).

### Comparison to previous single-molecule Pif1 studies

In addition to our study, Pif1 has been the subject of several other single-molecule investigations each touching upon different facets of the protein’s functional attributes. Results from SmFRET studies of *S. cerevisiae* Pif1 using a short dsDNA substrate with a 3′ ssDNA tail (35) provided evidence that Pif1 translocation along a 3′ ssDNA tail can be coupled to repetitive DNA looping activity, like the shuttling behavior reported for Srs2 and several other DNA helicases (35, 59, 60), with an estimated translocation velocity of 85 nt/sec at saturating ATP at 22 °C (35). Pif1 could also unwind a 31-bp RNA–DNA hybrid, although the unwinding rate was slow and required multiple attempts (35). Interestingly, Pif1 monomers were unable to unwind dsDNA duplex, but multiple Pif1 molecules acting upon the same dsDNA could promote unwinding (35). SmFRET studies have also shown that Pif1 can unwind G quadraplexes and does so in a series of steps to yield a fully unwound DNA strand (35). SmFRET studies with forked DNA substrates revealed both repetitive unwinding attempts and full substrate winding (61). These distinct modes of unwinding suggest that conformational transitions within Pif1 may regulate how it unwinds nucleic acids (61). Magnetic tweezer analysis of *S. cerevisiae* Pif1–mediated hairpin unwinding revealed unwinding rates of ∼50 nt/sec at low force up to 150 nt/sec at higher force and a processivity ranging from ∼25 bp to ∼200 bp at 22 °C (62). A second magnetic tweezer study of *S. cerevisiae* Pif1 yielded a translocation velocity of 140 nt/sec at 100 μM ATP on DNA extended by a force of 17 pN and an estimated maximum velocity of ∼220 nt/sec at saturating ATP at ∼23 °C (RT) (63). Notably, this study reported that most cases consisted of regular, unidirectional translocation events (∼90%), whereas a smaller fraction (∼10%) exhibited repetitive translocation events (63). Our conclusion that Pif1 can translocate for long distances on ssDNA also differs from early bulk biochemical analysis which suggested a highly distributive action of Pif1 on both ssDNA and dsDNA (32).

Our data show unidirectional Pif1 translocation on ssDNA that is much more rapid and translocates longer distances than reported in prior studies. One possible explanation for these differences is that no nucleic acid unwinding is taking place in our assays, so our data may indicate more rapid motion when the enzymes does not have to separate nucleic acid strands. This interpretation is consistent with the higher ATP hydrolysis activity we find for Pif1 with ssDNA *versus* dsDNA substrates. We also note that prior single-molecule studies measured translocation velocity at lower ATP concentrations (*e.g*., 20–100 μM), and lower reaction temperatures (22 °C) and used much smaller DNA substrates; these factors may all contribute to differences in results. Indeed, when analyzed at room temperature (∼20–22 °C), GFP–Pif1 translocated ∼70% more slowly (106 ± 66 nt/sec) and for shorter distances (11.8 ± 4.8 knt) in our assays compared to reactions performed at 30 °C (not shown).

## Conclusion

We have established a new single-molecule DNA curtain assay that allows us to directly visualize the translocation activity of GFP-tagged Pif1 on RPA-coated ssDNA. We have used this assay to probe the translocation characteristics of GFP-Pif1 and a large number of Pif1 mutant proteins harboring amino acid substitutions within the ssDNA-binding cleft. We anticipate that this assay to be adaptable for studying other aspects of Pif1 biophysics, for example its ability to unwinding nucleic acid structures such as G-quartets or RNA–DNA hybrids.

## Experimental procedures

### Protein expression and purification

RPA and Rad51 were purified as previously described (64). To generate GFP–Pif1, a DNA fragment encoding superfolder GFP was inserted at the N terminus of a gene for the Pif1 nuclear isoform (amino acid residues 40–859) encoded within a pRSF–Duet–1 vector (47, 65). Pif1 mutants were generated using an In-Fusion HD cloning plus kit (Takara Bio, Cat. No: 638910), and all mutants were verified by sequencing. Pif1 and GFP–Pif1 was expressed in *E. coli* Rosetta2 (DE3) cells (Novagen) by inducing 8 L of cell culture with 0.1 mM of isopropyl–1–thio β–D–galactopyranoside after the culture reached an absorbance of 0.6 to 0.8. The induced cells were grown for 16 °C for 18 h and then pelleted by centrifugation. The Pif1 and GFP–Pif1 purification protocol was adapted from a previously described procedure (47), with some minor modifications. The cell pellet was resuspended in 50 ml of buffer A (30 mM Tris–HCl [pH 7.5], 10% glycerol, 0.1% Triton X–100, and 2 mM DTT) containing 100 mM KCl, 1 mM phenylmethylsulfonyl fluoride, and one cOmplete protease inhibitor cocktail tablet (Roche, Cat. No. 05892953001). The cells were lysed by sonication, and the lysate was clarified by ultracentrifugation at 40K rpm for 45 min. The clarified lysate was loaded onto a Q–Sepharose FF column (2 ml; Cytiva Life Sciences, Cat. No. 17051010), and the flow–through was collected and then loaded onto a SP–Sepharose FF column (2 ml; Cytiva Life Sciences, Cat. No. 17072910). The column was washed with buffer A containing 150 mM KCl, and Pif1 was eluted in buffer A with a gradient of 150 to 650 mM KCl. The fractions containing the protein were pooled loaded onto a Ni–NTA column (1 ml; ThermoFisher Scientific, Cat. No. 88222). The Ni–NTA column was washed first with 30 ml of buffer A containing 1 M KCl and 15 mM imidazole and then again with 30 ml of buffer A containing 150 mM KCl, 1 mM ATP, 8 mM MgCl_2_, and 15 mM imidazole. The protein was then eluted with buffer A containing 150 mM KCl and 200 mM imidazole. The eluted protein was dialyzed overnight at 4 °C against buffer A containing 150 mM KCl. The dialyzed protein was then loaded onto a HiTrap SP HP column (1 ml; Cytiva Life Sciences, Cat. No. 29051324) and washed with buffer A containing 150 mM KCl. Pif1 was eluted in 150 to 650 mM KCl gradient in buffer A. Peaks were concentrated using Vivaspin 20 concentrator (30 kDa MWCO; Sartorius, Cat. No. VS2021) and stored in −80 °C.

### Helicase assays

For the helicase assay, we used an Alexa647–labeled DNA substrate containing 5′ ssDNA overhang. The substrate was prepared by annealing the following two oligonucleotides (Integrated DNA Technologies): 5′–Alexa647–GCT AGC AGT AGC CAG CAT CGA ACG TAC GAT CGG TAA CGT A–3’; 5′–CAT ATT TAA AAC ATG TTG GAT CCC AGC ACC AGA TTC AGC ATA CGT TAC CGA TCG TAC GTT CGA TGC TGG CTA CTG CTA GC–3’. For annealing, 15 μM (final concentration) of each oligonucleotide was mixed in a total reaction volume of 100 μl containing 10 mM Tris–HCl [pH 7.5], 50 mM NaCl, and 10 mM MgCl_2_. The mixture was heated to 95 °C for 2 min and then slowly cooled to 25 °C in a thermal cycler. The annealed product was purified on an 10% polyacrylamide gel run in TBE buffer. The annealed DNA was eluted from the gel using the crush and soak method in buffer containing 500 mM ammonium acetate plus 10 mM magnesium acetate, and the sample was incubated overnight at 16 °C. The sample was passed through a spin column (Takara Bio, Cat No: 740,609.50) to remove residual gel fragments and then further purified using a NucleoSpin Gel and PCR Clean-Up Kit (Takara Bio, Cat No: 740,609.50). Helicase assays were performed with the indicated amounts of Pif1 (or its variants), 10 nM of DNA substrate, and contained buffer with 35 mM Tris-HCl [pH 7.5], 1 mM DTT, 5 mM MgCl_2_, 60 mM KCl, 0.1 mg/ml BSA, 2 mM ATP, 20 mM creatine phosphate, 30 ng/μl creatine kinase, and 40 nM RPA. Reactions were incubated at 30 °C for 3 min and then terminated with the addition of equal volume of 2× stop solution containing 40 mM EDTA and 1% SDS. The reaction was then deproteinized with the addition of proteinase K (1 mg/ml) and incubated at 37 °C for 5 min. Reaction products were resolved in an 8% polyacrylamide gel in TBE buffer and imaged in a Typhoon FLA 9000 imager.

### ATP hydrolysis assays

The ATP hydrolysis assays were performed by incubating 10 nM Pif1 with either ssDNA (M13, 7249 nts; 1.5 μM nts final concentration; New England Biolabs, Cat. No. N4040S) or dsDNA (Curmid plasmid, 12,273 bps (64); 10 μM base pairs final concentration) and either RPA or Rad51 at the indicated concentrations in buffer containing 35 mM Tris–HCl [pH 7.5], 1 mM DTT, 5 mM MgCl_2_, 60 mM KCl, 2.5 mM ATP, and 0.1 mg/ml BSA and trace amount of [γ-^32^P]-ATP. Reactions were then incubated at 30 °C and aliquots removed at 5-, 10-, 20- and 30-min time points. The reactions were stopped by addition of an equal volume of 50 mM EDTA for a final EDTA concentration of 25 nM. The quenched reactions were then spotted onto TLC plates (Sigma–Aldrich, Cat No. Z740237–25EA) and resolved in 0.5 M LiCl and 1 M Formic acid. The TLC plates were dried and imaged with a Typhoon FLA 9000 imager (GE Healthcare). The amount of ATP hydrolysis was calculated from the ratio of hydrolyzed phosphate to unhydrolyzed ATP. The *k*_*obs*_ (in μM/s) was calculated from the slope of the initial linear region of the time-dependent ATP hydrolysis plot. To calculate *k*_*cat*_, different concentration of ATP (substrate) was used, and the *k*_*obs*_ was calculated for each substrate concentration. *K*_*cat*_ (in sec^–1^) was calculated by fitting the *k*_*obs*_ *versus* [ATP] plot with the Michaelis–Menten equation.

### ssDNA-binding assays

For the ssDNA-bindings assays, each specified Pif1 variants (0, 3.125, 6.25, 12.5, 25, 50 nM) was incubated with 5 nM of 40 nt 5′ Alexa–647-labeled ssDNA (5′–Alexa647–GCT AGC AGT AGC CAG CAT CGA ACG TAC GAT CGG TAA CGT A–3′) in buffer containing 35 mM Tris–HCl [pH 7.5], 1 mM DTT, 5 mM MgCl_2_, 60 mM KCl, and 0.1 mg/ml BSA. ATP was not included in the reactions. The reaction mixtures were incubated for 30 min at 30 °C and then mixed with an equal volume of 2× loading solution (20% glycerol plus 10 mM EDTA). The protein–DNA complex was resolved in an 8% polyacrylamide gel in TBE buffer and imaged in a Typhoon FLA 9000 imager (GE Healthcare). The ratio of the bound to unbound fraction was used to calculate the percentage of ssDNA which has protein bound for each protein concentration. The curve was fitted using specific enzyme-binding kinetics equation in Prism software to obtain the K_d_ for each Pif1 variant.

### Single-molecule assays

All experiments were conducted with a custom–built prism–type total internal reflection fluorescence microscope (Nikon) equipped with a 488–nm laser (Coherent Sapphire, 200 mW), a 561–nm laser (Coherent Sapphire, 200 mW), and two Andor iXon EMCCD cameras (54, 55). To prepare flow cells, chrome barriers were deposited on quartz microscope slides *via* e–beam lithography and thermal evaporation, as described (55, 66). Lipid bilayers were prepared with 91.5% DOPC (1,2–dioleoyl–sn–glycero–3–phosphocoline), 0.5% biotinylated–PE (1,2–dioleoyl–sn–glycero–3–phosphoethanolamine–N–(cap biotinyl), and 8% mPEG 2000–DOPE (1,2–dioleoyl–sn–glycero–3–phosphoethanoloamine–N–[methoxy(polyethylenegycol)–2000] (Avanti Polar Lipids, Inc., Cat. No. 850375P, 870273P and 880130P, respectively). Lipid bilayers were deposited in preformed flow chambers through sequential deposition of a lipid master mix in lipid buffer (20 mM Tris–Cl [pH 7.5], 100 mM NaCl).

The ssDNA substrate was generated by rolling circle replication using phi29 DNA polymerase with a biotinylated primer annealed to M13 circular ssDNA as a template (54, 55). The ssDNA was tethered to the bilayer through a biotin–streptavidin linkage in BSA buffer (40 mM Tris-HCl [pH 8.0], 2 mM MgCl_2_, 1 mM DTT, 0.2 mg/ml BSA), as described (54, 55). The ssDNA molecules were aligned at a flow rate of 0.8 ml/min in BSA buffer plus 0.1 nM RPA–mCherry. Once ssDNA molecules were aligned, 0.5 ml of 7 M urea was injected into the flow cell to further extend the ssDNA at the same flow rate. BSA buffer (40 mM Tris-HCl [pH 8.0], 2 mM MgCl_2_, 1 mM DTT, and 0.2 mg/ml BSA) containing 0.1 nM RPA-mCherry was then flushed through the sample chamber for 8 to 10 min rate of 0.8 ml/min. The flow cell was then washed with 4 ml of buffer P (35 mM Tris-HCl [pH 7.5], 1 mM DTT, 5 mM MgCl_2_, 60 mM KCl, 2.5 mM ATP, 0.2 mg/ml BSA) at a rate of 0.5 ml/min. After equilibrating with buffer P, GFP-Pif1 (0.5 nM) was injected through a 50 μl sample loop with continuous flow of buffer P (minus RPA-mCherry) at a rate of 0.2 ml/min, and the flow rate was kept constant until the end of the experiment. All single-molecule assays were conducted at 30 °C.

Image acquisition was initiated concurrently with GFP-Pif1 injection at a frame rate of one frame per 10 s for a total time of approximately 30 min. Data were collected with a 100–millisecond integration time, and the lasers were shuttered between images to minimizing photo–bleaching. Images were collected using Nikon software, and images were exported as individual Tag Image File Format (TIFF) images as described (54, 55). TIFF stacks were imported into ImageJ (Fiji). For two–color imaging, the two channels were first corrected for stage drift using the registration/translation function within Fiji (54). For each time course experiment, kymographs were generated from the TIFF image stacks by defining a 2–pixel wide region of interest along the axis of each individual ssDNA molecule, and these region of interests were extracted from each image within the TIFF stack (54). All slices corresponding to one ssDNA molecule were then aligned to yield a kymograph representing the entire experimental time course, and this process was repeated for each ssDNA molecule that was analyzed (54).

## Data availability

All data are contained in the article or available on request by contacting the corresponding author: ecg2108@cumc.columbia.edu.

## Supporting information

This article contains supporting information.

## Conflict of interest

The authors declare that they have no conflicts of interest with the contents of this article.

## Supporting information

Table S2

Figure S1 **Pif1 *versus* GFP–Pif1 ATPase activity with ssDNA *versus* dsDNA**. *A*, fraction of ATP hydrolyzed over time for WT Pif1 (unlabeled) in the presence or absence of ssDNA or dsDNA. *B*, fraction of ATP hydrolyzed over time for GFP-Pif1 (unlabeled) in the presence or absence of ssDNA or dsDNA. For both graphs, each experiment was conducted in triplicate and error bars represent the standard error of the mean. dsDNA, double-stranded DNA, ssDNA, single-stranded DNA.

Figure S2 **Gel assays for Pif1 mutants**. Examples of electrophoretic mobility shift assays (EMSA) for all Pif1 proteins and mutants in reactions with a 40-nt ssDNA substrate. See Table S1 for K_d_ values. Note that the panels for GFP-Pif1, Pif1 T301A, Pif1 K387A, and Pif1 E724A are reproduced from Figure 6 to allow for a side-by-side comparison with all other mutant proteins.

Figure S3 **All helicase assays for the Pif1 mutants**. Examples of gels showing the DNA unwinding activity for GFP-Pif1, and all the GFP-tagged Pif1 mutants. Minus Pif1 and heat denatured (H.D.) controls are also shown. Note that the gels for GFP-Pif1, Pif1 T301A, Pif1 K387A, and Pif1 E724A are reproduced from Figure 7 to allow for a side-by-side comparison with all other mutant proteins. See Table S1 for quantitation of the helicase activity assays.
