# Supplementary material for: Single-molecule visualization of Pif1 helicase translocation on single-stranded DNA
Source: J Biol Chem. 2023 May 11;299(6):104817. doi: 10.1016/j.jbc.2023.104817 (PMC10279920; doi:10.1016/j.jbc.2023.104817)
Supplement: Table S1 [file mmc1.docx]

|  | ATP hydrolysis  (V_max_ ; µM/sec) | ATP hydrolysis  (K_M_ ; mM) | ATP hydrolysis  (*k*_cat_ ; sec^–1^) | % Helicase activity  (3 min) | ssDNA binding  (K_d_; nM) |
| --- | --- | --- | --- | --- | --- |
| WT | 6.46 ± 1.26 | 0.49 ± 0.37 | 646.1 ± 125.7 | 79.27 ± 4.95 | 12.62 ± 3.81 |
| GFP–Pif1 | 6.41 ± 2.08  (P = 9.7x10^–1^) | 0.47 ± 0.62  (P = 1.36) | 640.9 ± ±208.4  (P = 9.7x10^–1^) | 73.23 ± 6.03  (P = 1.29) | 13.38 ± 4.80  (P = 0.72) |
| K264A | 0.00 ± 0.00  (P = 4.2x10^–7^) | N.D. | N.D. | N.D. | 12.06 ± 8.70  (P = 0.79) |
| G291P | 2.72 ± 0.42  (P = 2.9x10^–3^) | 0.76 ± 0.29  (P = 1.31) | 271.6 ± 41.6  (P = 2.9x10^–3^) | 0.29 ± 0.30  (P = 7.70x10^–42^) | 61.41 ± 15.80  (P = 1.0x10^–3^) |
| T301A | 1.78 ± 0.61  (P = 2.2x10^–4^) | 1.00 ± 0.83  (P = 1.21) | 177.8 ± 60.5  (P = 2.2x10^–4^) | 0.61 ± 0.39  (P = 1.73x10^–41^) | N.D. |
| H303A | 4.28 ± 3.90  (P = 8.2x10^–2^) | 0.87 ± 1.32  (P = 1.27) | 427.8 ± 389.8  (P = 8.2x10^–2^) | 39.57 ± 11.18  (P = 7.17x10^–10^) | 46.32 ± 5.96  (P = 0.0004) |
| S304A | 3.76 ± 1.35  (P = 3.1x10^–2^) | 0.72 ± 0.82  (P = 1.32) | 375.5 ± 134.8  (P = 3.1x10^–2^) | 17.52 ± 7.21  (P = 5.20x10^–25^) | 23.24 ± 4.01  (P = 0.03) |
| L310A | 2.79 ± 0.86  (P = 3.6x10^–3^) | 0.65 ± 0.63  (P = 1.34) | 279.3 ± 85.7  (P = 3.6x10^–3^) | 3.39 ± 1.56  (P = 1.73x10^–38^) | 27.84 ± 10.74  (P = 0.02) |
| K312A | 1.96 ± 0.28  (P = 3.7x10^–4^) | 0.38 ± 0.31  (P = 1.36) | 196.4 ± 28.1  (P = 3.7x10^–4^) | 29.95 ± 5.19  (P = 1.36x10^–15^) | 39.88 ± 1.45  (P = 0.005) |
| V385A | 4.75 ± 0.74  (P = 1.7x10^–1^) | 1.28 ± 0.54  (P = 1.03) | 475.2 ± 74.4  (P = 1.7x10^–1^) | 4.85 ± 2.82  (P = 5.85x10^–37^) | 28.45 ± 28.40  (P = 0.01) |
| K387A | 8.75 ± 1.15  (P = 9.3x10^–1^) | 0.54 ± 0.29  (P = 1.36) | 874.7 ± 115.2  (P = 9.3x10^–1^) | 76.00 ± 4.52  (P = 1.34) | 15.32 ± 4.76  (P = 0.23) |
| R465A | 2.00 ± 0.24  (P = 4.1x10^–4^) | 0.13 ± 0.17  (P = 1.30) | 199.8 ± 23.5  (P = 4.1x10^–4^) | 1.60 ± 1.28  (P = 2.09x10^–40^) | 43.45 ± 17.82  (P = 1.0x10^–3^) |
| N526A | 2.29 ± 0.32  (P = 9.4x10^–4^) | 1.18 ± 0.30  (P = 1.10) | 228.5 ± 32.3  (P = 9.4x10^–4^) | 3.16 ± 1.62  (P = 9.87x10^–39^) | 37.69 ± 10.65  (P = 2.0x10^–4^) |
| N533A | 3.06 ± 1.03  (P = 6.9x10^–3^) | 0.84 ± 0.65  (P = 1.28) | 306.4 ± 103.2  (P = 6.9x10^–3^) | 0.61 ± 0.40  (P = 1.73x10^–41^) | 54.53 ± 12.67  (P = 0.002) |
| S703A | 2.40 ± 0.68  (P = 1.3x10^–3^) | 2.37 ± 0.91  (P = 0.30) | 239.9 ± 68.3  (P = 1.3x10^–3^) | 2.15 ± 2.16  (P = 8.21x10^–40^) | 42.6 ± 9.25  (P = 1.0x10^–4^) |
| H705A | 1.68 ± 5.32  (P = 1.6x10^–4^) | 1.21 ± 7.33  (P = 1.08) | 167.6 ± 532.3  (P = 1.6x10^–4^) | 2.58 ± 1.53  (P = 2.38x10^–39^) | 32.23 ± 7.35  (P = 5.0x10^–4^) |
| F723A | 3.15 ± 0.67  (P = 8.4x10^–3^) | 1.58 ± 0.58  (P = 0.81) | 314.9 ± 66.6  (P = 8.4x10^–3^) | 1.88 ± 1.88  (P = 4.20x10^–40^) | 55.58 ± 18.54  (P = 2.0x10^–3^) |
| E724A | 4.55 ± 2.48  (P = 1.3x10^–1^) | 1.47 ± 1.37  (P = 0.89) | 454.5 ± 247.5  (P = 1.3x10^–1^) | 11.85 ± 4.50  (P = 4.59x10^–30^) | 18.69 ± 6.18  (P = 0.09) |

**Table S1.** Quantitation of Pif1 bulk biochemical data.

N.D. – Activity too weak to detect or quantitate.

Note – all P values correspond to comparison with the WT Pif1 (unlabeled) data sets.
