# Supplementary material for: Single-molecule visualization of Pif1 helicase translocation on single-stranded DNA
Source: J Biol Chem. 2023 May 11;299(6):104817. doi: 10.1016/j.jbc.2023.104817 (PMC10279920; doi:10.1016/j.jbc.2023.104817)
Supplement: Table S2 [file mmc2.docx]

|  | Velocity  (nucleotides/sec) | Processivity  (kilonucleotides) | N value | P value |
| --- | --- | --- | --- | --- |
| GFP–Pif1 | 348 ± 135 | 29.5 ± 14.1 | 200 | N.A. |
| K264A | N.D. | N.D. | ≥50 | N.A. |
| G291P | N.D. | N.D. | ≥50 | N.A. |
| T301A | N.D. | N.D. | ≥50 | N.A. |
| H303A | N.D. | N.D. | ≥50 | N.A. |
| S304A | N.D. | N.D. | ≥50 | N.A. |
| L310A | 73 ± 47 | 8.7 ± 4.1 | 154 | <1.0x10^–5^ |
| K312A | 223 ± 123 | 16.4 ± 8.6 | 197 | <1.0x10^–5^ |
| V385A | N.D. | N.D. | ≥50 | N.A. |
| K387A | 219 ± 112 | 19.9 ± 8.3 | 177 | <1.0x10^–5^ |
| R465A | N.D. | N.D. | ≥50 | N.A. |
| N526A | N.D. | N.D. | ≥50 | N.A. |
| N533A | N.D. | N.D. | ≥50 | N.A. |
| S703A | N.D. | N.D. | ≥50 | N.A. |
| H705A | N.D. | N.D. | ≥50 | N.A. |
| F723A | N.D. | N.D. | ≥50 | N.A. |
| E724A | 190 ± 74 | 14.8 ± 8.4 | 141 | <1.0x10^–5^ |

**Table S2.** Quantitation of Pif1 single molecule data.

N.D. – Activity not detected or negligible.

N.A. – Not applicable.

Note – all P values correspond to comparison with the GFP–Pif1 data sets.
