# Supplementary figures and images for: Single-molecule visualization of Pif1 helicase translocation on single-stranded DNA
Source: J Biol Chem. 2023 May 11;299(6):104817. doi: 10.1016/j.jbc.2023.104817 (PMC10279920; doi:10.1016/j.jbc.2023.104817)

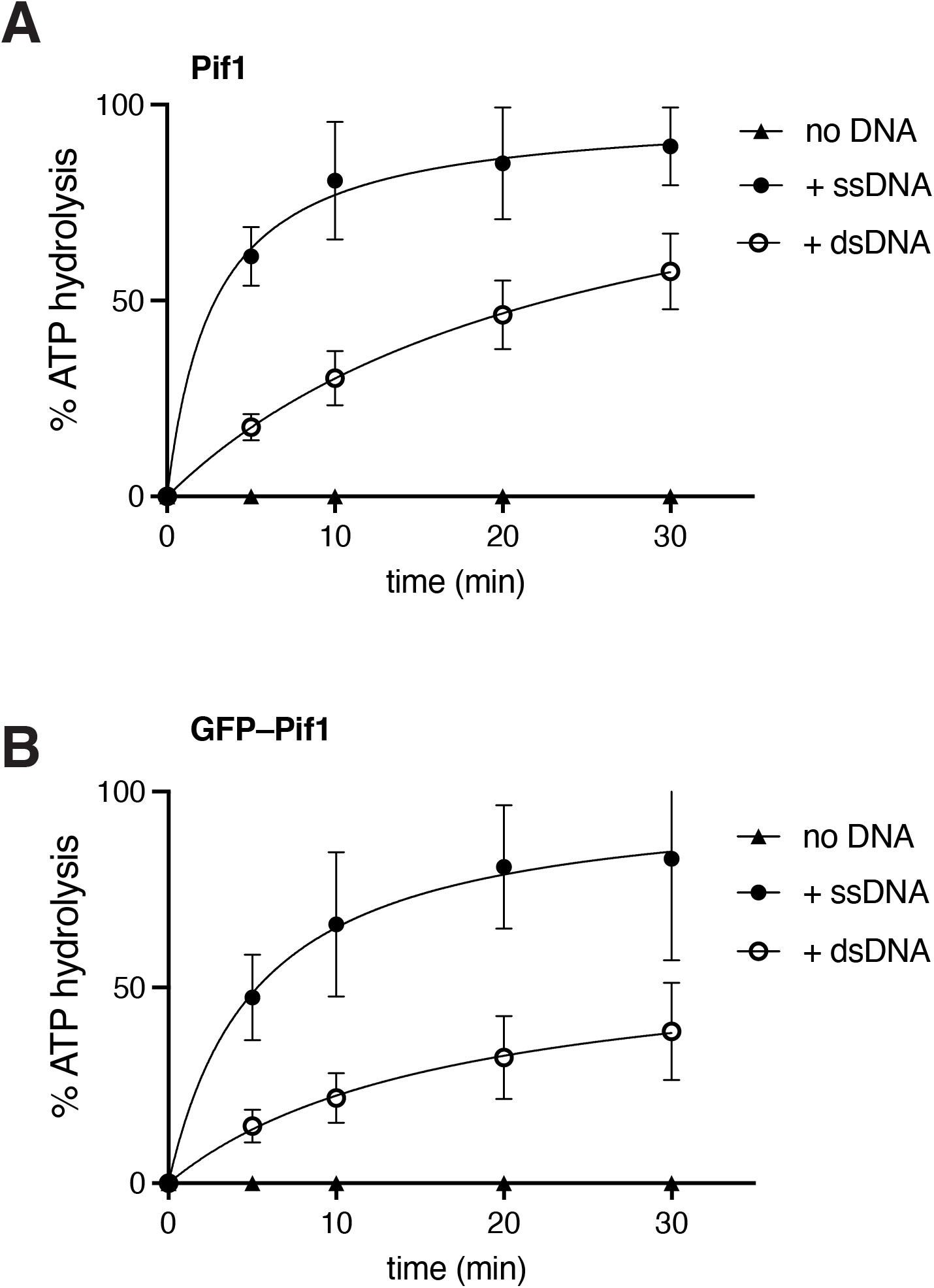

Supplement: Figure S1 — Pif1 versus GFP–Pif1 ATPase activity with ssDNA versus dsDNA. A, fraction of ATP hydrolyzed over time for WT Pif1 (unlabeled) in the presence or absence of ssDNA or dsDNA. B, fraction of ATP hydrolyzed over time for GFP-Pif1 (unlabeled) in the presence or absence of ssDNA or dsDNA. For both graphs, each experiment was conducted in triplicate and error bars represent the standard error of the mean. dsDNA, double-stranded DNA, ssDNA, single-stranded DNA. [file figs1.jpg]

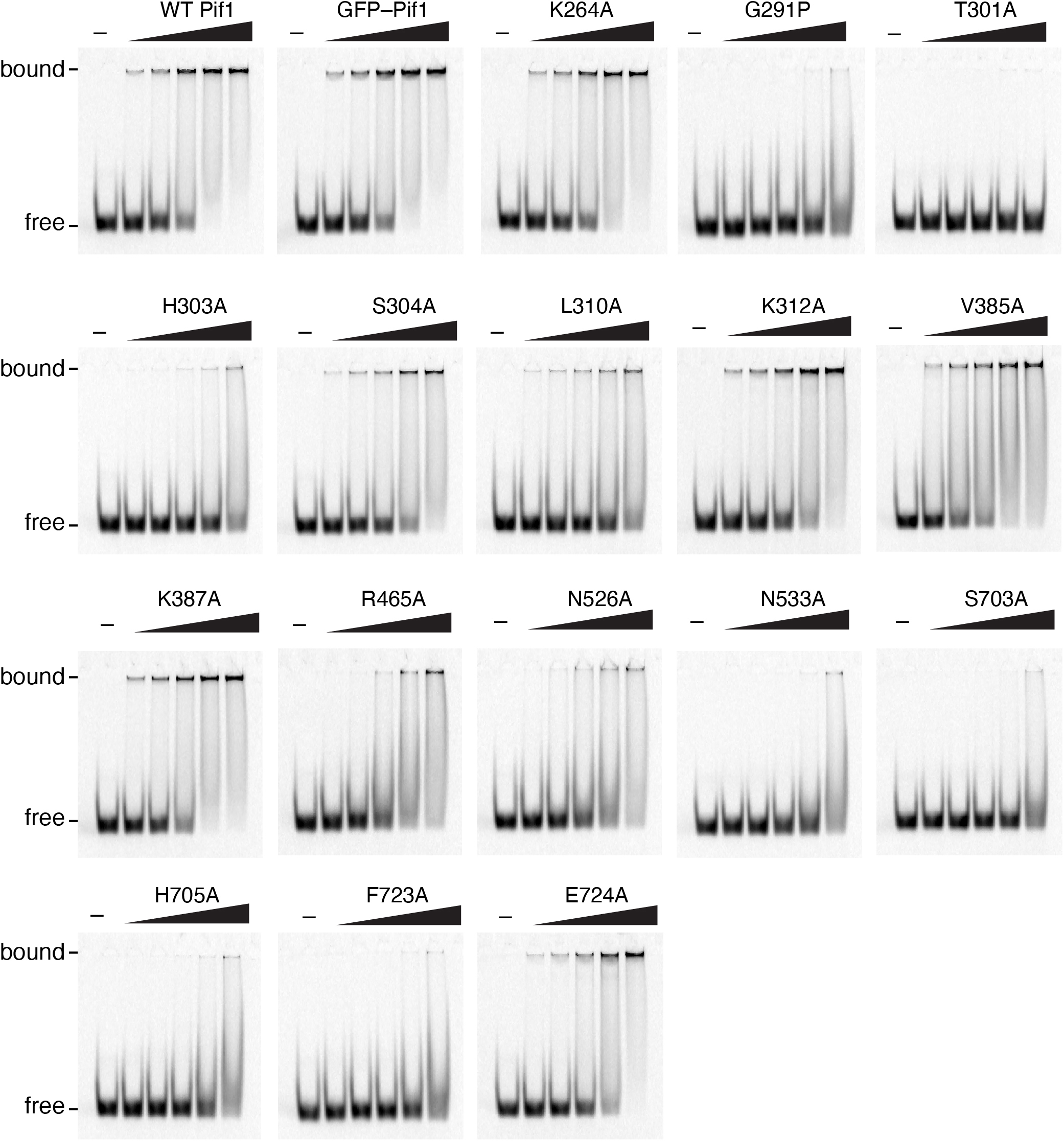

Supplement: Figure S2 — Gel assays for Pif1 mutants. Examples of electrophoretic mobility shift assays (EMSA) for all Pif1 proteins and mutants in reactions with a 40-nt ssDNA substrate. See Table S1 for Kd values. Note that the panels for GFP-Pif1, Pif1 T301A, Pif1 K387A, and Pif1 E724A are reproduced from Figure 6 to allow for a side-by-side comparison with all other mutant proteins. [file figs2.jpg]

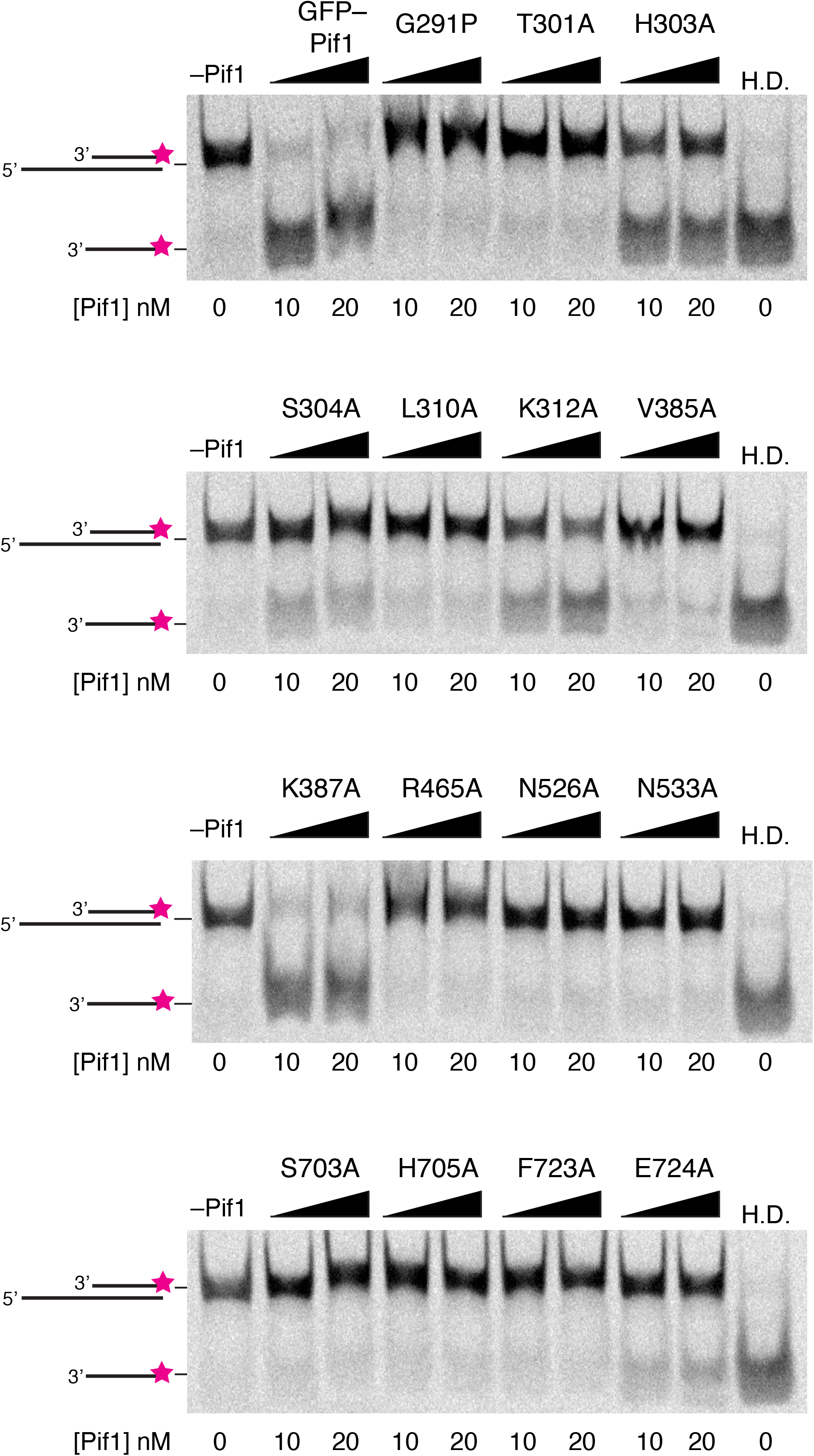

Supplement: Figure S3 — All helicase assays for the Pif1 mutants. Examples of gels showing the DNA unwinding activity for GFP-Pif1, and all the GFP-tagged Pif1 mutants. Minus Pif1 and heat denatured (H.D.) controls are also shown. Note that the gels for GFP-Pif1, Pif1 T301A, Pif1 K387A, and Pif1 E724A are reproduced from Figure 7 to allow for a side-by-side comparison with all other mutant proteins. See Table S1 for quantitation of the helicase activity assays. [file figs3.jpg]
